# The 22q11.2 Deletion Syndrome: A Gene Dosage Perspective

**DOI:** 10.1100/tsw.2006.317

**Published:** 2006-05-01

**Authors:** Antonio Baldini

**Affiliations:** Department of Pediatrics (Cardiology) and Molecular and Human Genetics, Baylor College of Medicine, Houston, TX 77030 and CEINGE Institute, University Federico II, Naples, Italy

**Keywords:** gene dosage, Tbx1, DiGeorge syndrome, 22q11.2DS

## Abstract

The 22q11.2 deletion/DiGeorge syndrome is a relatively common “genomic” disorder that results from heterozygous deletion of a 3-Mbp segment of chromosome 22. Of the more than 30 genes deleted in this syndrome, *TBX1* is the only one that has been found to be mutated in some patients with a phenotype that is very similar to that of patients with the full deletion, suggesting that *TBX1* haploinsufficiency is a major contributor to the syndromes phenotype. Multi- and single-gene mouse models have provided a considerable amount of information about the consequences of decreased and increased dosage of the genomic region (and in particular of the *Tbx1* gene) on mouse embryonic development. Modified alleles of *Tbx1*, as well as conditional ablation strategies have been utilized to map *in vivo* the tissues and developmental stages most sensitive to gene dosage. These experiments have revealed substantially different sensitivity to gene dosage in different tissues and at different times, underlying the importance of the developmental context within which gene dosage reduction occurs.

## 22q11.2DS/DIGEORGE SYNDROME IS A CLINICALLY IMPORTANT EXAMPLE OF A DISORDER CAUSED BY GENE DOSAGE ALTERATION

Gene dosage alterations are an important cause of congenital abnormalities and embryonic or neonatal lethality. Entire chromosomes (monosomies or trisomies) or portions of them (segmental aneuploidies) can be lost or be present in excess. Among the segmental aneuploidies, microdeletion syndromes are relatively frequent, and among those, 22q11.2 deletion syndrome (22q11.2DS) is thought to be the most common. This chromosomal deletion is the most common cause of DiGeorge and velocardiofacial syndromes. Clinical features of these syndromes have been extensively reviewed[1,2,3,4,5,6]. As with many segmental aneuploidies, the clinical presentation can be variable (in penetrance and expressivity of individual clinical symptoms) even within the same family[7] or among monozygotic twins[8,9], indicating that genetic and nongenetic factors can contribute to variability. However, to date, there are no reports of 22q11.2 deletion without any associated abnormality.

The deletion is similar in most patients, indeed greater than 90% of them have the same 3-Mbp deletion[10,11,12]. Low copy repeats at the extremities of the deleted region are thought to represent a substrate for aberrant recombination, as in other microdeletion syndromes[10,11,13,14]. Duplications of the same region, resulting from the same recombination event, have also been found in patients with a less characteristic and consistent phenotype[15,16]. Thus, both deletion and duplication of the region can lead to phenotypic abnormalities.

The 3-Mbp 22q11.2 deletion spans 28 genes, while a less common, 1.5-Mbp deletion encompasses about 21 genes (Fig. 1). However, clinical reports have not been able to distinguish clearly or phenotypically patients with the two types of deletions[17]. Given the relatively small number of patients with the smaller deletion, and given the variability of the phenotype, it is difficult to draw definitive conclusions however.

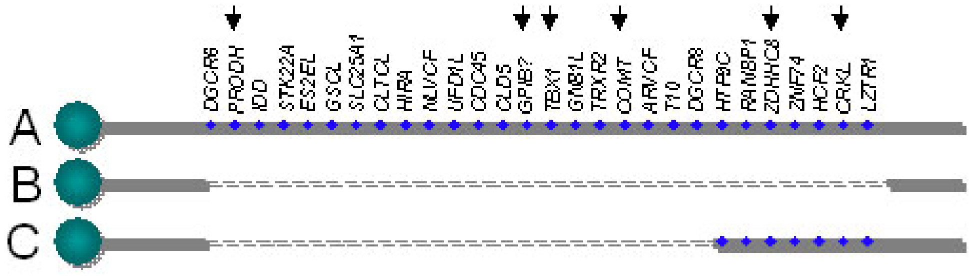

**FIGURE 1.** Genes eliminated by the two most common deletions in 22q11.2DS patients. (A) Normal human chromosome, (B) deletion present in approximately 90% of the patients, (C) deletion present in approximately 10% of the patients. Genes indicated by arrows are cited in the text. The drawing is not to scale.

The 22q11.2DS is most likely to be a contiguous gene syndrome, a condition in which the haploinsufficiency of different genes contributes to different clinical features. For example, platelet abnormalities are most likely contributed by *GP1BB* deletion, while there are several candidate genes for the behavioral and psychiatric disorders (*PRODH*, *COMT*, *ZDHHC8*, and possibly others[18]). However, the most characteristic clinical findings of the syndrome, which are those related to developmental defects of the embryonic pharyngeal apparatus, are caused by haploinsufficiency of *TBX1*[19,20]. The phenotypic complex related to abnormal pharyngeal development includes thymic, parathyroid, cardiovascular, and craniofacial anomalies. The developmental importance of *Tbx1* was delineated in the mouse in 2001[21,22,23] and, thus, it became the major candidate gene for the syndrome. In 2003, Yagi et al.[19] identified *TBX1* mutations, including point mutations, in patients with typical 22q11.2DS phenotype, but without deletion. At least one of those mutations was later shown to cause *TBX1* loss of function because it prevents the TBX1 protein from localizing to the nucleus, where it exerts its function as a transcription factor[20].

Genes in the deleted region may contribute to the phenotype not only through a contiguous gene syndrome mechanism, i.e., by causing a particular aspect of the phenotype, but also by modifying, or in some way interacting with, the *TBX1* haploinsufficiency phenotype. The gene *CRKL* is included in the large 3-Mbp deletion (but not in the 1.5-Mbp deletion) and the *Crkl*$^{-/-}$ mouse mutant phenotype includes abnormalities related to maldevelopment of the pharyngeal apparatus[24]. Crosses between *Tbx1* and *Crkl* mutants have revealed that *Crkl* mutation strongly enhances the *Tbx1* haploinsufficiency phenotype, indicating an interaction between the two genes[25]. These results are somewhat at odds with medical genetics data that have not identified striking clinical differences between patients with the 1.5- and 3-Mbp deletions. However, as mentioned above, these clinical studies should be extended before drawing definitive conclusions. It would also be desirable to generate a multigene deletion mouse encompassing all the mouse homologs of genes in the 22q11.2 3-Mbp deletion, as the region may harbor not only enhancers, but also suppressors, of the *Tbx1* mutant phenotype.

## TESTING GENE DOSAGE SENSITIVITY IN THE MOUSE

The initial mouse modeling work generated a deletion (*Df1*) and a duplication (*Dp1*) of part of the region harboring genes homologous to those deleted in 22q11.2DS[26]. *Df1*/+ mice had cardiovascular abnormalities, thymic and parathyroid hypoplasia, as well as learning and behavioral abnormalities[26,27,28]. A larger deletion (*Lgdel*/+) was reported later with a similar phenotype, although the behavioral phenotype of this mutant has not been reported[21].

Important questions concerning multigene deletion mutants are (1) what is the effect of the rearrangement on neighboring genes and (2) is the level of expression of heterozygously deleted genes compensated for by up-regulation of the nondeleted alleles? To answer these questions, Prescott et al.[29] have performed transcriptome analysis of mid-gestation *Df1*/+ embryos using microarrays. Results indicated that all the deleted genes tested had reduced expression (approximately 50%), hence providing no evidence for dosage compensation. In addition, the rearrangement appeared not to have any effect on the expression of neighboring genes. Thus, the rearrangement caused no transcriptional disregulation *in cis*. Furthermore, despite the fact that the rearrangement involved 20 genes, the disruption of the transcriptome was relatively mild.

The consequences of increased copy number of the *Df1* or *Lgdel* genomic regions are less studied. Mice with three or four copies of the *Df1* region (*Dp1*/+ and *Dp1*/*Dp1* mice) were reported to be normal[26], even though three copies of the region deleted in 22q11.2DS cause phenotypic abnormalities in humans. In the light of new knowledge about the tissues and developmental times that are critical for the morphogenesis of the pharyngeal apparatus, it would be of interest to re-examine *Dp1/Dp1* embryos and phenotype them molecularly, for example, through transcriptome analysis of the appropriate tissues at the appropriate developmental times. Comparisons of transcriptomes of *Df1*/+, +/+, *Dp1*/+, and *Dp1/Dp1* animals, i.e., animals that carry one, two, three, and four copies of the genomic region, respectively, may identify genes the expression of which correlates with the number of copies of the *Df1* region. Despite the reported lack of abnormalities in mice carrying four copies of the *Df1* region, transgenic mice carrying multiple copies (eight to ten) of a human BAC harboring *TBX1* and three other genes (corresponding to a subsegment of the *Df1* region), were shown to have abnormalities reminiscent of the *Tbx1* haploinsufficiency phenotype[21,30]. This phenotype could be partially corrected by crossing these transgenic mice with *Tbx1* mutants, demonstrating that at least some of the phenotypic abnormalities observed in multicopy transgenic mice are due to extra copies of the *TBX1* gene[30]. It would be of interest to establish whether the overexpression phenotype obtained with the human gene can also be obtained with the mouse gene in multicopy transgenics.

Although multigene mutants represent more accurate models of segmental aneuploidies, single gene mutants offer considerable advantages for research because they are less complex and because one can engineer specialized alleles for detailed analysis of gene function. Mouse and human embryonic development is clearly sensitive to reduced dosage of the *Tbx1* gene, and *Tbx1*$^{+/-}$ animals present with a distinctive phenotype, mostly aortic arch and great artery defects, that results from hypoplasia of the fourth pharyngeal arch arteries (PAA)[31]. This phenotype is milder than that of most 22q11.2DS patients. *Tbx1*$^{-/-}$ animals have severe abnormalities of the cardiac outflow tract and of other derivatives of the pharyngeal apparatus and they die soon after birth[22,32]. Thus, the heterozygous phenotype is too mild and the null phenotype too severe for further analysis of the sensitivity of developmental processes to *Tbx1* dosage. Further information has been obtained by modifying the *Tbx1* allele so as to reduce mRNA dosage. Hu et al. and Xu et al.[33,34] have generated hypomorphic alleles of *Tbx1* and have shown that different organs and, presumably, different developmental processes, have different sensitivity to *Tbx1* mRNA dosage. In particular, the craniofacial phenotype (including cleft palate) appeared to be less sensitive than the cardiovascular phenotype. These results provide a new perspective to the dosage sensitivity phenomenon, albeit a predictable one: Dosage sensitivity is a characteristic of the context within which the gene product functions. Thus, it is important to establish the tissue and developmental time in which the gene dosage sensitive process occurs. The most dosage-sensitive developmental process is growth and/or remodeling of the fourth PAA. Defects of this process occur not only in *Tbx1*$^{+/-}$ embryos, which express 50% of the wild type *Tbx1* mRNA, but also in $Tbx1^{neo/+}$ embryos, which express 60% of WT mRNA ($Tbx1^{neo}$ is a hypomorphic allele producing about 10% mRNA of the wild type allele). Conditional deletion experiments using Cre drivers recombining in different tissues of the pharyngeal apparatus have shown that heterozygous deletion of *Tbx1* in pharyngeal epithelia (ectoderm and endoderm) is sufficient to cause fourth PAA hypoplasia[35]. Ablation of the gene with a Cre driver recombining predominantly in the endoderm failed to reproduce this phenotype[36]. Together, these results suggest that the critical tissue may be the ectoderm, although positive confirmation that deletion in the ectoderm is sufficient to cause the fourth PAA phenotype must be obtained. Because ectodermal cells do not contribute directly to the fourth PAA, Tbx1 must have a cell-nonautonomous effect on the formation, growth, or remodeling of these arteries, possibly by regulating the expression of genes coding for extracellular signaling molecules. Systematic analysis of the temporal requirement for *Tbx1* for various developmental processes in the mouse has shown that fourth PAA development requires full *Tbx1* dosage in early development, beginning around embryonic day (E) 8, and lasting for approximately 24 h[37]. Such an early requirement coincides with the onset of *Tbx1* expression in the ectoderm and in other tissues[35]. Hence, ectodermal function of *Tbx1* at E8–E9 may hold the key to our understanding of the molecular mechanisms underlying the most Tbx1-dosage sensitive processes of mouse development.

## POSSIBLE MECHANISMS UNDERLYING GENE DOSAGE SENSITIVITY

Tbx1 is a transcription factor of the T-box family. T-box proteins have critical functions in development of vertebrates and invertebrates, and several of the T-box encoding genes are haploinsufficient in humans and mice[38]. Current data suggest that Tbx1 works as a transcription activator[33,34,39]. Generally speaking, proteins participating in macromolecular complexes (like transcription factors) are more likely to be dosage sensitive[40]. However, this does not easily explain tissue- and time-specific gene dosage sensitivity because, presumably, Tbx1 needs interactors wherever and whenever its function is required. Tbx1 may participate to different types of complexes; for example, it may interact with other proteins to activate specific targets or it may participate in chromatin remodeling complexes and thereby have a broader transcriptional effect. Both scenarios have been reported for other T-box proteins (e.g., for Tbx5[41,42,43]), but not as yet for Tbx1. Thus, the phenotypic consequences of altered gene dosage may be different in different tissues and may be anatomically more or less obvious. The effect that has been more frequently reported for Tbx1 is one on cell proliferation[33,34,37], but it is unknown whether a cell proliferation defect causes the haploinsufficiency phenotype. Nevertheless, modulation of cell proliferation could explain many phenotypic findings of $Tbx1^{-/-}$ and conditional mutants, and it could also explain phenotypic variability, especially if there is a linear correlation between Tbx1 protein dosage and the rate of proliferation. For example, Tbx1 may regulate, in a dosage-dependent manner, Fgf8 and Fgf10 expression[33,34,44] that could convey a proproliferation signal.

There is also an alternative explanation for tissue- and time-specific dosage sensitivity, the hint of which comes from an apparently unlikely correlation. The most Tbx1-dosage-sensitive developmental process, namely fourth PAA growth or remodeling, is also the one that requires *Tbx1* at an early developmental time point. In contrast, the least dosage-sensitive process (among those studied), namely palate closure, is the one that requires *Tbx1* at a late developmental point[37]. Simulations of stochastic gene expression predict that the loss of one copy of a gene has a more deleterious effect, in terms of reduced accumulation of gene product, during the process of activation of gene transcription, rather than during a steady-state phase of expression[45]. In other words, gene haploinsufficiency may cause significant delay in the achievement of a critical level of gene product after the signals for gene activation have been deployed. Thus, developmental processes that require *Tbx1* near the time of gene activation are more likely to be affected than those that require the gene product at a later time (Fig. 2). This might explain why fourth PAA development, which requires Tbx1 in the ectoderm around the time when the gene is normally activated, is so sensitive to Tbx1 dosage.

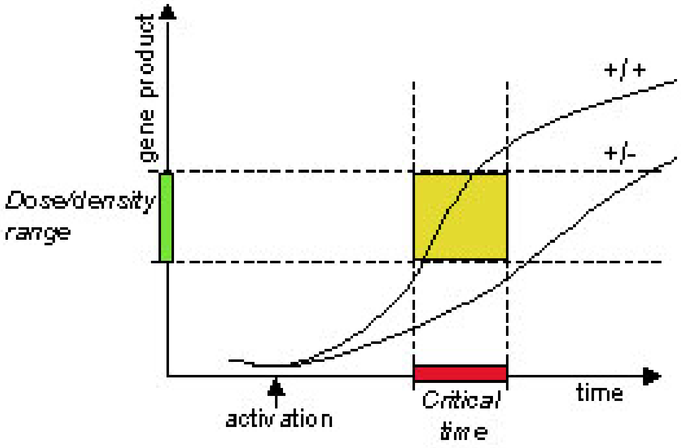

**FIGURE 2.** Schematic drawing illustrating a model of integration of temporal and dosage requirements for a given developmental process in a specific cell population. The assumption, supported by experimental data, is that the gene product (e.g., Tbx1) has only a limited time to effect its function. The "dose/density range" refers to the minimum concentration of gene product in a cell population required for the developmental process to occur normally. The deletion of one copy of the gene may increase the time necessary for the gene product to reach a particular concentration, as predicted by models of stochastic gene expression[45].

## CONCLUDING REMARKS

The multigene deletion models generated so far have provided critical information that led to the identification of *Tbx1*, the gene that is sufficient to cause a 22q11.2DS-like phenotype in humans and mice when mutated. However, none of the deletions produced in mice so far encompass all the genes deleted in 22q11.2DS patients; thus, we do not know if other genes may contribute, positively or negatively, to penetrance and expressivity of the *Tbx1* haploinsufficiency phenotype. Indeed, recent data have shown that *Crkl* heterozygous mutation can enhance the *Tbx1* haploinsufficiency phenotype in mice (*Crkl* is not included in any of the available multigene-deletion models)[25]. The generation and characterization of full-deletion models should afford better comparisons between the mouse and human disorders. Perhaps, morphological phenotyping should be integrated with transcriptome analysis in animals with different copies of the genomic region (reduced and in excess) to identify genes the expression of which correlate with copy number. These may be candidate effectors of the gene dosage phenotype.

It is predictable, however, that considerable effort will continue to be dedicated to the analysis of Tbx1 function. Of relevance to the scope of this review, future research into the mechanisms of gene dosage sensitivity should have two main objectives.

The first objective would be to identify proteins interacting directly with the Tbx1 protein. Interactions with protein partners in transcriptional or chromatin remodeling complexes is likely to be a critical source of Tbx1 dosage sensitivity. To date, no Tbx1 interactor has been reported. It will be of interest to test whether reduced dosage of the interactor may rescue the haploinsufficiency phenotype by re-establishing correct stoichiometry. This may happen if the complex is simple (e.g., heterodimer), but less likely to work if Tbx1 contributes to large multiprotein complexes.

The second objective would be to study the dynamics of *Tbx1* gene expression in the precise developmental context (time and tissue) in which dosage sensitivity is more pronounced. This may be technically challenging and may require some creative experimental approaches, but may provide evidence, and measure, of the predicted delay in gene activation caused by heterozygous mutations in the appropriate context.

In conclusion, clinical relevance, developmental importance, and availability of several mutant alleles make *Tbx1* an excellent model for testing dosage sensitivity mechanisms *in vivo*.

## ACKNOWLEDGMENTS

Research in the author's laboratory is supported by grants from the NIH, from COFIN2005, and from the Italian Telethon.

## References

1. Perez, E. and Sullivan, K.E. (2002) Chromosome 22q11.2 deletion syndrome (DiGeorge and velocardiofacial syndromes). *Curr. Opin. Pediatr.* **14,** 678–683.
2. Yamagishi, H. (2002) The 22q11.2 deletion syndrome. *Keio J. Med.* **51,** 77–88.
3. Digilio, M.C., Marino, B., Cappa, M., Cambiaso, P., Giannotti, A., and Dallapiccola, B. (2001) Auxological evaluation in patients with DiGeorge/velocardiofacial syndrome (deletion 22q11.2 syndrome). *Genet. Med.* **3,** 30–33.
4. Botto, L.D., May, K., Fernhoff, P.M., Correa, A., Coleman, K., Rasmussen, S.A., Merritt, R.K., O'Leary, L.A., Wong, L.Y., Elixson, E.M., Mahle, W.T., and Campbell, R.M. (2003) A population-based study of the 22q11.2 deletion: phenotype, incidence, and contribution to major birth defects in the population. *Pediatrics* **112,** 101–107.
5. Matsuoka, R., Kimura, M., Scambler, P.J., Morrow, B.E., Imamura, S., Minoshima, S., Shimizu, N., Yamagishi, H., Joh-o, K., Watanabe, S., Oyama, K., Saji, T., Ando, M., Takao, A., and Momma, K. (1998) Molecular and clinical study of 183 patients with conotruncal anomaly face syndrome. *Hum. Genet.* **103,** 70–80.
6. Murphy, K.C. (2005) Annotation: velo-cardio-facial syndrome. *J. Child Psychol. Psychiatry* **46,** 563–571.
7. Iascone, M.R., Vittorini, S., Sacchelli, M., Spadoni, I., Simi, P., and Giusti, S. (2002) Molecular characterization of 22q11 deletion in a three-generation family with maternal transmission. *Am. J. Med. Genet.* **108,** 319–321.
8. Vincent, M.C., Heitz, F., Tricoire, J., Bourrouillou, G., Kuhlein, E., Rolland, M., and Calvas, P. (1999) 22q11 deletion in DGS/VCFS monozygotic twins with discordant phenotypes. *Genet. Couns.* **10,** 43–49.
9. Yamagishi, H., Ishii, C., Maeda, J., Kojima, Y., Matsuoka, R., Kimura, M., Takao, A., Momma, K., and Matsuo, N. (1998) Phenotypic discordance in monozygotic twins with 22q11.2 deletion. *Am. J. Med. Genet.* **78,** 319–321.
10. Saitta, S.C., Harris, S.E., Gaeth, A.P., Driscoll, D.A., McDonald-McGinn, D.M., Maisenbacher, M.K., Yersak, J.M., Chakraborty, P.K., Hacker, A.M., Zackai, E.H., Ashley, T., and Emanuel, B.S. (2004) Aberrant interchromosomal exchanges are the predominant cause of the 22q11.2 deletion. *Hum. Mol. Genet.* **13,** 417–428.
11. McDermid, H.E. and Morrow, B.E. (2002) Genomic disorders on 22q11. *Am. J. Hum. Genet.* **70,** 1077–1088.
12. Shaikh, T.H., Kurahashi, H., Saitta, S.C., O'Hare, A.M., Hu, P., Roe, B.A., Driscoll, D.A., McDonald-McGinn, D.M., Zackai, E.H., Budarf, M.L., and Emanuel, B.S. (2000) Chromosome 22-specific low copy repeats and the 22q11.2 deletion syndrome: genomic organization and deletion endpoint analysis. *Hum. Mol. Genet.* **9,** 489–501.
13. Edelmann, L., Pandita, R.K., Spiteri, E., Funke, B., Goldberg, R., Palanisamy, N., Chaganti, R.S., Magenis, E., Shprintzen, R.J. and Morrow, B.E. (1999) A common molecular basis for rearrangement disorders on chromosome 22q11. *Hum. Mol. Genet.* **8,** 1157–1167.
14. Shaffer, L.G. and Lupski, J.R. (2000) Molecular mechanisms for constitutional chromosomal rearrangements in humans. *Annu. Rev. Genet.* **34,** 297–329.
15. Emanuel, B.S. and Shaikh, T.H. (2001) Segmental duplications: an 'expanding' role in genomic instability and disease. *Nat. Rev. Genet.* **2,** 791–800.
16. Ensenauer, R.E., Adeyinka, A., Flynn, H.C., Michels, V.V., Lindor, N.M., Dawson, D.B., Thorland, E.C., Lorentz, C.P., Goldstein, J.L., McDonald, M.T., Smith, W.E., Simon-Fayard, E., Alexander, A.A., Kulharya, A.S., Ketterling, R.P., Clark, R.D., and Jalal, S.M. (2003) Microduplication 22q11.2, an emerging syndrome: clinical, cytogenetic, and molecular analysis of thirteen patients. *Am. J. Hum. Genet.* **73,** 1027–1040.
17. Rauch, A., Zink, S., Zweier, C., Thiel, C.T., Koch, A., Rauch, R., Lascorz, J., Huffmeier, U., Weyand, M., Singer, H., and Hofbeck, M. (2005) Systematic assessment of atypical deletions reveals genotype-phenotype correlation in 22q11.2. *J. Med. Genet.* **42,** 871–876.
18. Paylor, R. and Lindsay, E. (2006) Mouse models of 22q11 deletion syndrome. *Biol. Psychiatry*, in press.
19. Yagi, H., Furutani, Y., Hamada, H., Sasaki, T., Asakawa, S., Minoshima, S., Ichida, F., Joo, K., Kimura, M., Imamura, S.-I., Kamatani, N., Momma, K., Takao, A., Nakazawa, M., Shimizu, N., and Matsuoka, R. (2003) Role of TBX1 in human del22q11.2 syndrome. *Lancet* **362,** 1366–1373.
20. Stoller, J.Z. and Epstein, J.A. (2005) Identification of a novel nuclear localization signal in Tbx1 that is deleted in DiGeorge syndrome patients harboring the 1223delC mutation. *Hum. Mol. Genet.* **14,** 885–892.
21. Merscher, S., Funke, B., Epstein, J.A., Heyer, J., Puech, A., Min Lu, M.M., Xavier, R.J., Demay, M.B., Russell, R.G., Factor, S., Tokooya, K., St. Jore, B., Lopez, M., Pandita, R.K., Lia, M., Carrion, D., Xu, H., Schorle, H., Kobler, J.B., Scambler, P.J., Wynshaw-Boris, A., Skoultchi, A.I., Morrow, B.E., and Kucherlapati, R. (2001) TBX1 is responsible for cardiovascular defects in velo-cardio-facial/DiGeorge syndrome. *Cell* **104,** 619–629.

22. Jerome, L.A. and Papaioannou, V.E. (2001) DiGeorge syndrome phenotype in mice mutant for the T-box gene, *Tbx1. Nat. Genet*. **27,** 286–291.
23. Lindsay, E.A., Vitelli, F., Su, H., Morishima, M., Huynh, T., Pramparo, T., Jurecic, V., Ogunrinu, G., Sutherland, H.F., Scambler, P.J., Bradley, A., and Baldini, A. (2001) Tbx1 haploinsufficieny in the DiGeorge syndrome region causes aortic arch defects in mice. *Nature* **410,** 97–101.
24. Guris, D.L., Fantes, J., Tara, D., Druker, B.J., and Imamoto, A. (2001) Mice lacking the homologue of the human 22q11.2 gene CRKL phenocopy neurocristopathies of DiGeorge syndrome. *Nat. Genet*. **27,** 293–298.
25. Guris, D.L., Duester, G., Papaioannou, V.E., and Imamoto, A. (2006) Dose-dependent interaction of Tbx1 and Crkl and locally aberrant RA signaling in a model of del22q11 syndrome. *Dev. Cell* **10,** 81–92.
26. Lindsay, E.A., Botta, A., Jurecic, V., Carattini-Rivera, S., Cheah, Y.-C., Rosenblatt, H.M., Bradley, A., and Baldini, A. (1999) Congenital heart disease in mice deficient for the DiGeorge syndrome region. *Nature* **401,** 379–383.
27. Taddei, I., Morishima, M., Huynh, T., and Lindsay, E.A. (2001) Genetic factors are major determinants of phenotypic variability in a mouse model of the DiGeorge/del22q11 syndromes. *Proc. Natl. Acad. Sci. U. S. A.* **98,** 11428–11431.
28. Paylor, R., McIlwain, K.L., McAninch, R., Nellis, A., Yuva-Paylor, L.A., Baldini, A., and Lindsay, E.A. (2001) Mice deleted for the DiGeorge/velocardiofacial syndrome region show abnormal sensorimotor gating and learning and memory impairments. *Hum. Mol. Genet.* **10,** 2645–2650.
29. Prescott, K., Ivins, S., Hubank, M., Lindsay, E., Baldini, A., and Scambler, P. (2005) Microarray analysis of the Df1 mouse model of the 22q11 deletion syndrome. *Hum. Genet.* **116,** 486–496.
30. Liao, J., Kochilas, L., Nowotschin, S., Arnold, J.S., Aggarwal, V.S., Epstein, J.A., Brown, M.C., Adams, J., and Morrow, B.E. (2004) Full spectrum of malformations in velo-cardio-facial syndrome/DiGeorge syndrome mouse models by altering Tbx1 dosage. *Hum. Mol. Genet.* **13,** 1577–1585.
31. Lindsay, E.A. (2001) Chromosomal microdeletions: dissecting del22q11 syndrome. *Nat. Rev. Genet.* **2,** 858–868.
32. Vitelli, F., Morishima, M., Taddei, I., Lindsay, E.A. and Baldini, A. (2002) Tbx1 mutation causes multiple cardiovascular defects and disrupts neural crest and cranial nerve migratory pathways. *Hum. Mol. Genet.* **11,** 915–922.
33. Hu, T., Yamagishi, H., Maeda, J., McAnally, J., Yamagishi, C., and Srivastava, D. (2004) Tbx1 regulates fibroblast growth factors in the anterior heart field through a reinforcing autoregulatory loop involving forkhead transcription factors. *Development* **131,** 5491–5502.
34. Xu, H., Morishima, M., Wylie, J.N., Schwartz, R.J., Bruneau, B.G., Lindsay, E.A., and Baldini, A. (2004) Tbx1 has a dual role in the morphogenesis of the cardiac outflow tract. *Development* **131,** 3217–3227.
35. Zhang, Z., Cerrato, F., Xu, H., Vitelli, F., Morishima, M., Vincentz, J., Furuta, Y., Ma, L., Martin, J.F., Baldini, A., and Lindsay, E. (2005) Tbx1 expression in pharyngeal epithelia is necessary for pharyngeal arch artery development. *Development* **132,** 5307–5315.
36. Arnold, J.S., Werling, U., Braunstein, E.M., Liao, J., Nowotschin, S., Edelmann, W., Hebert, J.M., and Morrow, B.E. (2006) Inactivation of Tbx1 in the pharyngeal endoderm results in 22q11DS malformations. *Development* **133,** 977–987.
37. Xu, H., Cerrato, F., and Baldini, A. (2005) Timed mutation and cell-fate mapping reveal reiterated roles of Tbx1 during embryogenesis, and a crucial function during segmentation of the pharyngeal system via regulation of endoderm expansion. *Development* **132,** 4387–4395.
38. Naiche, L.A., Harrelson, Z., Kelly, R.G., and Papaioannou, V.E. (2005) T-box genes in vertebrate development. *Annu. Rev. Genet.* **39,** 219–239.
39. Ataliotis, P., Ivins, S., Mohun, T.J., and Scambler, P.J. (2005) XTbx1 is a transcriptional activator involved in head and pharyngeal arch development in Xenopus laevis. *Dev. Dyn.* **232,** 979–991.
40. Veitia, R.A. (2002) Exploring the etiology of haploinsufficiency. *Bioessays* **24,** 175–184.
41. Lickert, H., Takeuchi, J.K., Von Both, I., Walls, J.R., McAuliffe, F., Adamson, S.L., Henkelman, R.M., Wrana, J.L., Rossant, J., and Bruneau, B.G. (2004) Baf60c is essential for function of BAF chromatin remodelling complexes in heart development. *Nature* **432,** 107–112.
42. Bruneau, B.G., Nemer, G., Schmitt, J.P., Charron, F., Robitaille, L., Caron, S., Conner, D.A., Gessler, M., Nemer, M., Seidman, C.E., and Seidman, J.G. (2001) A murine model of Holt-Oram syndrome defines roles of the T-box transcription factor Tbx5 in cardiogenesis and disease. *Cell* **106,** 709–721.
43. Hiroi, Y., Kudoh, S., Monzen, K., Ikeda, Y., Yazaki, Y., Nagai, R., and Komuro, I. (2001) Tbx5 associates with Nkx2-5 and synergistically promotes cardiomyocyte differentiation. *Nat. Genet*. **28,** 276–280.
44. Vitelli, F., Taddei, I., Morishima, M., Meyers, E.N., Lindsay, E.A., and Baldini, A. (2002) A genetic link between Tbx1 and fibroblast growth factor signaling. *Development* **129,** 4605–4611.
45. Cook, D.L., Gerber, A.N., and Tapscott, S.J. (1998) Modeling stochastic gene expression: implications for haploinsufficiency. *Proc. Natl. Acad. Sci. U. S. A.* **95,** 15641–15646.
